# Correction: Bar-Sela et al. Cannabis Consumption Used by Cancer Patients during Immunotherapy Correlates with Poor Clinical Outcome. *Cancers* 2020, *12*, 2447

**DOI:** 10.3390/cancers14081957

**Published:** 2022-04-13

**Authors:** Gil Bar-Sela, Idan Cohen, Salvatore Campisi-Pinto, Gil M. Lewitus, Lanuel Oz-Ari, Ayellet Jehassi, Avivit Peer, Ilit Turgeman, Olga Vernicova, Paula Berman, Mira Wollner, Mor Moskovitz, David Meiri

**Affiliations:** 1 Cancer Center, Emek Medical Center, 21 Yitzhak Rabin Blvd, Afula 1834111, Israel; idan5161@gmail.com (I.C.); olga_vr@clalit.org.il (O.V.); 2 Bruce Rappaport Faculty of Medicine, Technion-Israel Institute of Technology, Haifa 320002, Israel; lanuel.fuchs@gmail.com; 3 The Laboratory of Cancer Biology and Cannabinoid Research, Department of Biology, Technion-Israel Institute of Technology, Haifa 320003, Israel; campisi.pinto@gmail.com (S.C.-P.); lgil@technion.ac.il (G.M.L.); bermansh@gmail.com (P.B.); 4 Statistic Unit, Emek Medical Center, Afula 1834111, Israel; ayelletaj@gmail.com; 5 Division of Oncology, Rambam Health Care Campus, Haifa 320002, Israel; a_peer@rambam.health.gov.il (A.P.); i_turgeman@rambam.health.gov.il (I.T.); m_wollner@rambam.health.gov.il (M.W.); m_moskovitz@rambam.health.gov.il (M.M.)

## Error in Table

In the original article [1], there was a mistake in Table 1 as published. Typing errors were made regarding demographics and medical conditions data. The corrected Table 1 appears below. 

The authors apologize for any inconvenience caused and state that the scientific conclusions are unaffected. The original article has been updated.

## Figures and Tables

**Table 1:** Demographics and medical conditions.

Characteristics	Cannabis Non-Users *N* = 68	Cannabis Users *N* = 34	*p*-Value
Age in years median (range)	69 (18–92)	66 (37–85)	
Gender—*N* (%)			
Female	22 (32.4)	10 (29.5)	
Male	46 (67.6)	24 (70.5)	0.9399
Performance Status Eastern Cooperative Oncology Group (ECOG)—*N* (%)			
1≤	55 (80.8)	24 (70.5)	0.3568
≥2	13 (19.1)	10 (29.4)	0.3568
Chronic diseases per patient—*N* (%)			
0	22 (32.3)	13 (22.0)	0.7124
1	16 (23.5)	7 (20.5)	0.9332
2 or more	30 (44.1)	14 (41.1)	0.9437
Background diseases—*N* (%)			
Chronic heart disease	18 (26.4)	5 (14.7)	0.2762
Diabetes	17 (25.0)	6 (17.6)	0.5576
High blood pressure	34 (50.0)	13 (34.1)	0.3612
Chronic obstructive pulmonary disease (COPD)	9 (13.2)	3 (8.8)	1
Hyperlipidemia	23 (33.8)	7 (20.5)	0.2491
Other	2 (2.9)	0 (0.0)	1
Type of malignancy—*N* (%)			
Non-small cell lung cancer	37 (54.4)	20 (58.8)	0.8325
Melanoma	25 (36.7)	9 (26.4)	0.414
Renal cell carcinoma	4 (5.8)	2 (5.8)	1
Other	2 (2.9)	3 (8.8)	1
Main site of metastasis—*N* (%)			
Brain	12 (17.6)	8 (13.2)	0.6593
Lungs	39 (57.3)	23 (67.6)	0.4303
Liver	13 (19.1)	11 (32.3)	0.2157
Immunotherapy given as—*N* (%)			
First line of treatment	31 (45.5)	8 (23.5)	0.05178
Second line of treatment or more	37 (54.4)	26 (76.4)	0.05178
Checkpoint therapy—*N* (%)			
Anti PD1: Pembrolizumab or Nivolumab	47 (69.1)	29 (85.2)	0.127
Ipilimumab and Nivolumab	16 (23.5)	4 (11.7)	0.2517
Anti PDL-1: Durvalumab or Atezolizumab	5 (7.3)	1 (2.9)	1
